# Through the eyes of the Andean bear: Camera collar insights into the life of a threatened South American Ursid

**DOI:** 10.1002/ece3.70304

**Published:** 2024-12-04

**Authors:** Ruthmery Pillco Huarcaya, Andrew Whitworth, Norma Mamani, Mark Thomas, Elias Condori

**Affiliations:** ^1^ Asociación para la Conservación de la Cuenca Amazónica (ACCA) Cusco Peru; ^2^ Universidad Nacional de San Antonio Abad del Cusco (UNSAAC) Cusco Peru; ^3^ Osa Conservation Washington DC USA; ^4^ Osa Conservation Puerto Jiménez Puntarenas Costa Rica

**Keywords:** cannibalism, Manu, spectacled bear, technology, telemetry, Ursidae

## Abstract

Due to Andean bears' propensity for inhabiting challenging environments and terrain, their wild ecology remains poorly understood, especially when compared to other members of the Ursidae family. In one of the steepest, wettest regions of the Andes, the Kosñipata Valley of southeastern Peru, we attached and retrieved camera‐borne collars on three wild free‐ranging Andean bears. From just one longer term camera collar deployed on a single individual over a period of 4 months, we observed a variety of rare or previously undocumented natural history observations. These include courtship and mating behaviors, social interactions with conspecifics, novel dietary items of previously unrecorded fruit consumption, cannibalism, potential infanticide, the sole documented case of primate consumption, and evidence of geophagy. The wealth of novel natural history insights gained from just 4 months of camera collar data of this poorly studied species has elucidated numerous avenues warranting further investigation.

## Graphical abstract

This study used a camera‐equipped GPS collar on a male Andean bear in southeastern Peru, revealing rare behaviors like courtship, novel diets, and geophagy. These findings from the Kosñipata Valley provide valuable insights into the poorly understood ecology of Andean bears and suggest many areas for further research.

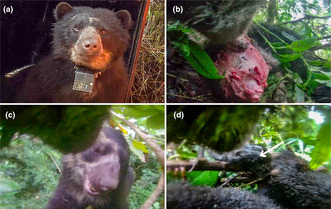

## 1 INTRODUCTION

Monitoring and understanding the behavior and ecology of elusive and threatened wildlife is crucial for their conservation. In recent years, camera‐borne collars have been increasingly used to address data gaps in ecology and natural history for a number of wildlife species, especially in the global north (Vuillaume et al., 2021). These innovative systems have been successfully deployed on various species of bear, including brown bears (*Ursus arctos*), Asiatic black bears (*Ursus thibetanus*), and polar bears (*Ursus maritimus*), providing valuable insights into their ecology and habitat use. For brown bears, camera collars were used to field‐test the collars, document the accuracy of feeding site‐focused surveys as a method for determining bear habitat use and food habits and determine the kill rates of ungulates (Bowersock et al., 2015; Brockman et al., 2017). In Asiatic black bears, collars recorded the activities of wild bears during the mating season and were used to compare bear food habits with fecal analysis (Naganuma et al., 2021; Tezuka et al., 2022). For polar bears, a tri‐axial accelerometer attached to a camera collar was used to identify behaviors and daily energy expenditure (Pagano et al., 2017, 2024; Pagano & Williams, 2019).

One elusive species of bear, however that to our knowledge has never previously been fitted with camera collar‐borne technology, is the Andean bear (*Tremarctos ornatus*). At least no published works could be found using this technology on this species. Andean bears are the only bear species found on the South American continent and are vulnerable according to the International Union for Conservation of Nature Red List of Threatened Species. They face significant threats, including habitat loss and fragmentation, possible range shifts due to climate change, and poaching, often related to conflict associated with crops and livestock, which has significantly impacted their population across their range (Velez‐Liendo & García‐Rangel, 2017). Due to their propensity to inhabit challenging environments and terrain, such as montane cloud forests and high Andean grasslands, studying Andean bears has proven to be particularly difficult, especially in understanding their diet patterns and social interactions. As a result, there are significant gaps in our understanding of their ecology and behavior compared with other members of the Ursidae family (Velez‐Liendo & García‐Rangel, 2017).

We aimed to enhance our knowledge of the ecology and natural history of wild Andean bears by utilizing animal‐borne GPS collars. We deployed three collar‐based cameras on three free‐ranging wild Andean bears. In a preliminary test, we successfully deployed and retrieved National Geographic's Crittercam system (a short‐term camera system on a separate release to the satellite collar and with no integration) on two relatively young male bears. We later attached and retrieved the first longer‐term camera‐borne GPS satellite collar (an integrated system to align camera footage with location and accelerometer data) to another male bear. Specifically, we wanted to understand the potential of this technology to provide valuable insights into the feeding habits, habitat use, and social interactions of this elusive species.

First, we expected to see bears mostly feeding upon bromeliads and wild blueberries, as this is what we have predominantly detected in over 207 scats collected from the region over the past 2 years. However, we also predicted that the cameras would pick up other food species that might be difficult to detect from traditional scat surveys, due to digestion processes, an outcome observed in other surveys using camera‐borne collars in wild ursids (Tezuka et al., 2022). Finally, we expected that the camera would record intraspecific and interspecific behaviors, which are extremely challenging to gather from static camera traps and direct observations, especially in such a challenging landscape.

## 2 METHODS

### 2.1 Study area

Our research was carried out on the eastern slope of the Andes in the Kosñipata Valley, situated in the buffer zone of Manu National Park, southeastern Peru. Our primary research station for captures was Wayqecha Biological Station (−13.174800, −71.587200). The Kosñipata Valley is part of the Tambopata–Manu wet spot (Killeen et al., 2007), with elevations ranging from 500 to 4000 m. The region's vegetation includes moist tropical rainforest in the lowlands, tropical montane cloud forest at mid‐elevations (lower and upper montane cloud forest), and Puna (Andean grassland) above the treeline (Rapp & Silman, 2012). This section of the Andes exhibits three seasonal periods: the wet season from November to March, the dry season from May through July, and the transitional months of April, August, September, and October (Rapp & Silman, 2012).

The Andean grassland terrain is steeply rolling and mountainous, where the Puna vegetation is dominated by the family Poaceae, with grasses from the genus *Jarava*, *Calamagrostis*, and *Festuca*, and patches of shrubs and herbs of the Asteraceae, the Ericaceae, and terrestrial bromeliads of the genus *Puya*. The upper montane forests include numerous species of trees, shrubs, lianas, epiphytes, and herbs. The canopy often reaches 10–25 m in height. Tree species from the families *Asteraceae*, *Cunoniaceae*, *Melastomataceae*, *Rosaceae*, *Rubiaceae*, and bamboo in the genus *Chusquea* dominate the upper montane cloud forest. Trees are covered with vascular and nonvascular epiphytes, including bryophytes, ferns, *Bromeliaceae*, *Ericaceae*, and *Orchidaceae* (Gibbon et al., 2010).

The topography of tropical lower montane forests is steep and rugged, with forests 10–30 m high with a dense understory. Trees have a lower epiphytic mass compared to those in upper cloud forests, with far fewer bromeliads. The forests may be even taller on gentler slopes, with trees reaching up to 40 m. Tree species from the *Lauraceae*, *Moraceae*, *Melastomataceae*, *Euphorbiaceae*, and *Cyatheaceae* families dominate these forests (Farfan–Rios et al., 2015).

### 2.2 Capturing and collaring

Collared bears were captured using an Iznachi trap baited with meat (Castellanos, Jackson, et al., 2016). The Iznachi trap was connected to a satellite trap transmitter TT5 (Vectronic Aerospace, Germany), which sent notifications daily and alerted the team via email immediately when the trap was triggered. Following established physical and chemical containment protocols, the trapped bears were immobilized with ketamine (4 mg/kg), dexmedetomidine (0.02 mg/kg), midazolam (0.2 mg/kg), and atipamezole (0.2 mg/kg) (Arias‐Bernal & Yarto‐Jaramillo, 2018). Both capture and immobilization procedures were reviewed, and permits were approved by the National Forest and Wildlife Service of Peru (SERFOR) Ethical Committee (Permits RD No. D000066‐2022‐MIDAGRI‐SERFOR‐DGGSPFFS‐DGSPFS). A veterinary evaluation of specimen health was conducted, including blood, saliva, samples, and morphometric measurements. Age was estimated based on tooth wear and gum condition. This was judged by eye using veterinarian experience following methods described by Shimoinaba and Oi (2015).

As a preliminary pilot test, we successfully deployed and retrieved National Geographic's Crittercam system on two relatively young male bears—one between 6 and 7 years old, weighing 59 kg, and another between 4 and 5 years old, weighing 33 kg. Both were captured in the reserve surrounding Wayqecha Biological Station. This short‐term camera system was set to record videos every 15 min, for 3‐min lengths, between the hours of 06:00 and 17:00 local time/and the programmed radio drop‐off was set to release the camera after 3 and 4 days. These camera systems are not integrated into the collar's location data, meaning that retrieval is carried out with a separately attached VHF transmitter to the camera. The camera was attached to the battery casing of a traditional GPS satellite wildlife collar (VERTEX LITE; Vectronic Aerospace, Germany). Note that the Crittercam footage data here are used to reference the pilot work conducted before the deployment of the longer term integrated camera collar system. This pilot study served to define later programming schedules for the built‐in camera collar and to analyze the feasibility for us to recover the device in this specific landscape and terrain. To ensure data comparability with the findings of the long‐term camera collar, we used only the first 15 s from each of our video from the Crittercams. The footage is not utilized in the description of our findings but serves as a reference for the diet description.

**FIGURE 1:**
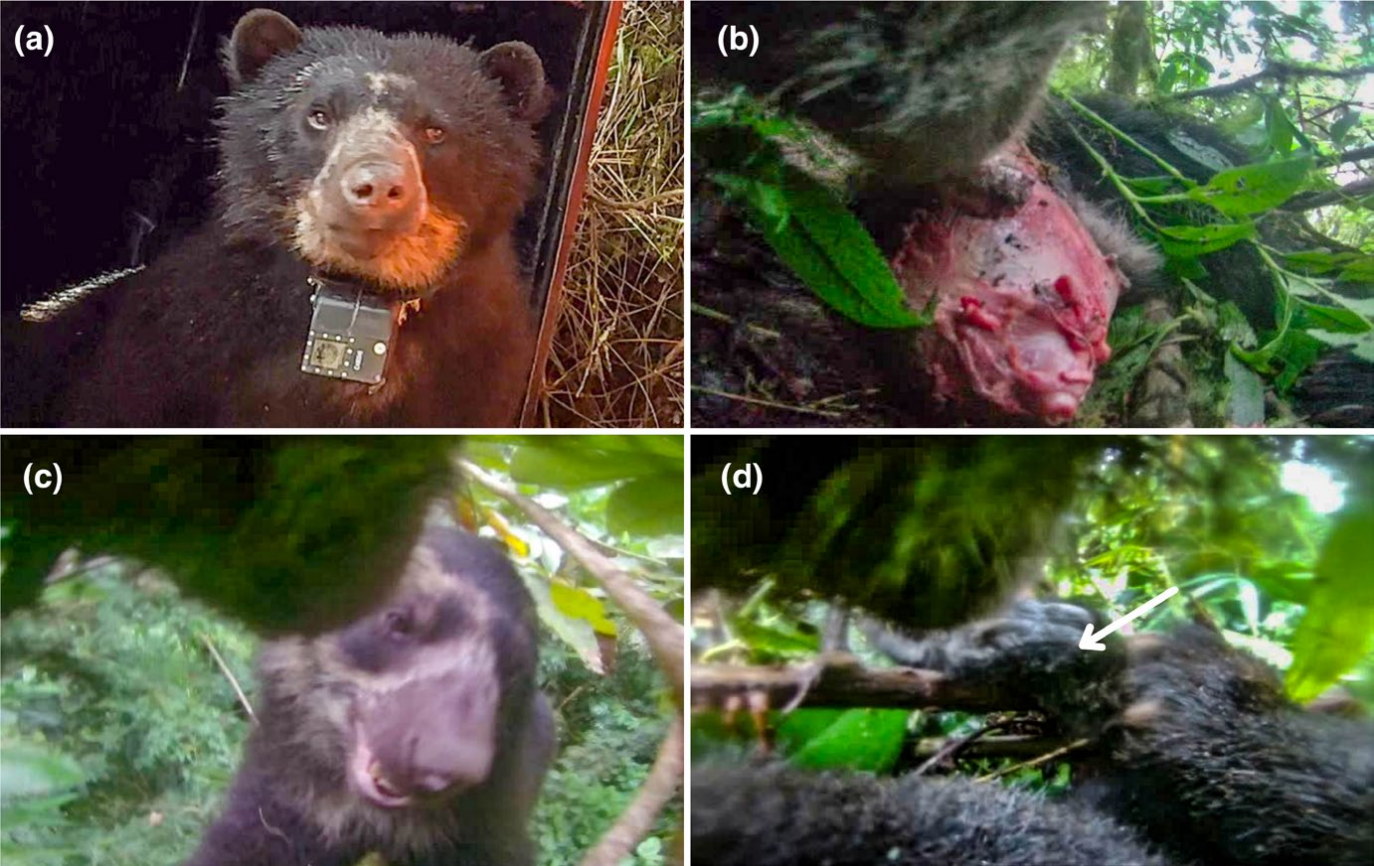
Notable events captured by the longer term camera collar. (a) Andean bear fitted with the VERTEX camera collar (photograph by Ian Rock). (b–d) Still captures from videos retrieved from the camera: (b) feeding on a bear cub carcass on November 16, 2023, at 4:58 pm. (c) Playfully interacting in the canopy with a female bear on March 17, 2024, at 7:00 am. (d) Feeding on the leg of a woolly monkey in the canopy on March 11, 2024, at 3:00 pm. The arrow is pointing to the hand of the primate.

We later attached and retrieved a longer‐term camera‐borne GPS satellite collar (an integrated system to align camera footage with location and accelerometer data to another male bear 7–8 years old, weighing 52.7 kg—see Figure 1) captured in the Bosque Nublado Private Conservation Area, nearby Wayqecha Biological Station. We used a GPS‐Iridium collar with an integrated camera (VERTEX PLUS Collar, Vectronic Aerospace, Germany) and a radio drop‐off. The collar was programmed to take GPS positions every hour, and the camera was programmed to take 15‐s high‐definition (1920 × 1080) video clips every hour during daylight hours (from 06:00 to 17:00 local time in Peru).

## 3 RESULTS AND DISCUSSIONS

The longer‐term collar recorded 1505 separate video clips (totaling 6 h and 27 min of footage over a 4‐month period ‐ November 14, 2023 to March 19, 2024). These bear‐perspective clips provide novel insights into new aspects in relation to the life of the Andean bear.

Despite the longer term data coming from a single individual, this unique perspective footage uncovered a variety of rarely, or previously undocumented, natural history‐related events. These include courtship and mating, social interactions with other bears, diet discoveries in terms of novel fruit consumption, cannibalism, potential infanticide and the only documented case of feeding on a sympatric primate, and finally, evidence of geophagy in Andean bears.

### 3.1 Diet, cannibalism, and geophagy

We observed a variation in dietary patterns, potentially linked to altitude in relation to the elevational distribution of available plant food resources. From the pilot Crittercam deployment footage of the two bears captured and released in the high Andean grassland and tree line for example, Bear 1 recorded 12 feeding events overall, with 11 on bromeliads (from 9 min of footage over 3 days, ranging from 2340 to 3600 m a.s.l.), while Bear 2 recorded 16 feeding events, 4 on bromeliads and 12 on wild blueberries (from 12 min of footages over 4 days, ranging from 3260 to 3700 m a.s.l.). This is in line with what we expected to see given that our findings from processing over 207 bear scats collected from within the region indicate that bears in the high Andean grasslands primarily consume both terrestrial and epiphytic bromeliads (*Puya, Guzmania, Tillandsia, and Pitcairnia* spp.—detected in 69% of recovered scats) and wild blueberries (*Vaccinium, Pernettya, Siphonandra, Gaultheria* spp.—detected in 17% of recovered scats).

In contrast, the bear collared with the longer term integrated camera ranged from just 1204 to 2944 m a.s.l. (average elevation of 1492 m a.s.l.) in the lower and upper montane cloud forests, never entering the high‐elevation grasslands during the 4 months. This individual ate bromeliads on just 23 occasions from a total of 338 feeding events. Overall, approximately 21 different species of plants could be identified being consumed from footage during the 4‐month period. These were predominantly stems from the Maranthaceae and Araceae families and the buds of the Arecaceae palms. The diet also includes a significant amount of *Ficus*, *Porcelia*, and *Cecropia*. Notably, *Porcelia ponderosa* (Annonaceae—custard apple family), *Urera lianoides* (Urticaceae—stinging nettle family), and *Siparuna sff. aspera* (Siparunaceae family) are all newly recorded species for Andean bear diet.

The footage from the longer‐term integrated collared bear also displayed clear recordings of 14 events where the bear was foraging for invertebrates both on the ground and in the canopy. However, it was challenging to identify specific prey through video footage. Despite this, the frequent presence of insect exoskeletons and larvae in collected scats underscores the significant role of invertebrates in the Andean bear diet (32 scats with insects). This limitation of camera collars highlights the need for complementary methods, such as genetic processing of scat samples. Tezuka et al. (2022) conducted a similar study comparing the dietary habits of the Asiatic black bear using traditional fecal analysis and camera collar footage. They also found that both methods are complementary tools, each with its advantages and disadvantages. Video analyses excelled in identifying food items, such as leaves or mammals, that are often crushed beyond recognition in fecal samples. However, camera collars are less effective at capturing food items that are infrequently or quickly ingested.

Through 2 years of on‐foot field surveys along the elevational gradient (550–3850 m a.s.l.), we collected 207 scats and remains of consumed plants of Andean bears. However, only 49 of these samples were collected below 2900 m a.s.l. This can be attributed to the dense understory, humid, rainy, and warmer elevational habitat, where scats are harder to locate and degrade faster. This means that diet from scat data is limited for forest‐dwelling bears at lower elevations. Bear‐perspective camera footage, such as the data we collected here, can therefore provide valuable dietary insight to complement traditional scat assessments.

One of the most surprising dietary items that was observed in the video clips was the consumption of a woolly monkey (*Lagothrix cana*) on the 10th and 11th of March. The first nine videos show the bear with the monkey carcass on the ground, which he later takes into the tree canopy, where the hand of the monkey can clearly be seen. It is uncertain if this incidence represents a predation or a scavenging event. The previous videos before he was observed feeding show him foraging on the ground, so this is more suggestive that he foraged the carcass of a deceased monkey. The only other published record of any bear species feeding on a nonhuman primate is of an Asiatic black bear consuming the carcass of a golden snub‐nosed monkey in China (Huang et al., 2014). While most bear species are associated with eating meat as part of an omnivorous diet, Andean bears are suggested to focus largely on fruits and plants (Figueroa, 2013; García‐Rangel, 2012), but large bears in particular, have been detected killing livestock, and foraging on animal carcasses (Pisso‐Florez et al., 2021; Rojas‐VeraPinto et al., 2022). Nonetheless, to our knowledge, feeding on a sympatric living nonhuman primate has never previously been documented in Andean bears until now.

In addition to primate meat as part of the collared bear's diet, he also displayed cannibalism. From November 16 to November 18, 2023, the video clips show the collared bear feeding on a dead bear cub. This activity took place on the ground. He started with the head and finished with the stomach. During these days, the bear also foraged on plants. While we cannot be certain that this represents an incident of infanticide, it is clear that the videos show a much smaller cub‐looking bear, and it is just a month prior to his courtship and mating events with the female in December. A second cannibalism event was observable in the footage on January 1, 2024. After a long journey on December 31, crossing the river and Kosñipata valley road, into the side of Manu National Park, the collared bear was observed on the morning of January 1 in the canopy, feeding on what appeared to be the carcass of a small bear. This appears to be a foraged part eaten carcass. He spent the day feeding on the carcass and continued his journey the following day. The only reports on cannibalism in Andean bears come from Ecuador (Castellanos, Pillajo, & Torres, 2016). Cannibalism has been commonly documented in other ursids, including polar bears, Asiatic black bears, brown bears, and American black bears (Allen et al., 2022), where cannibalism in these species seems most frequently associated with infanticide. The first record captured on the camera of the collared male bear here is suggestive of the first recorded case of Andean bear infanticide.

**FIGURE 2:**
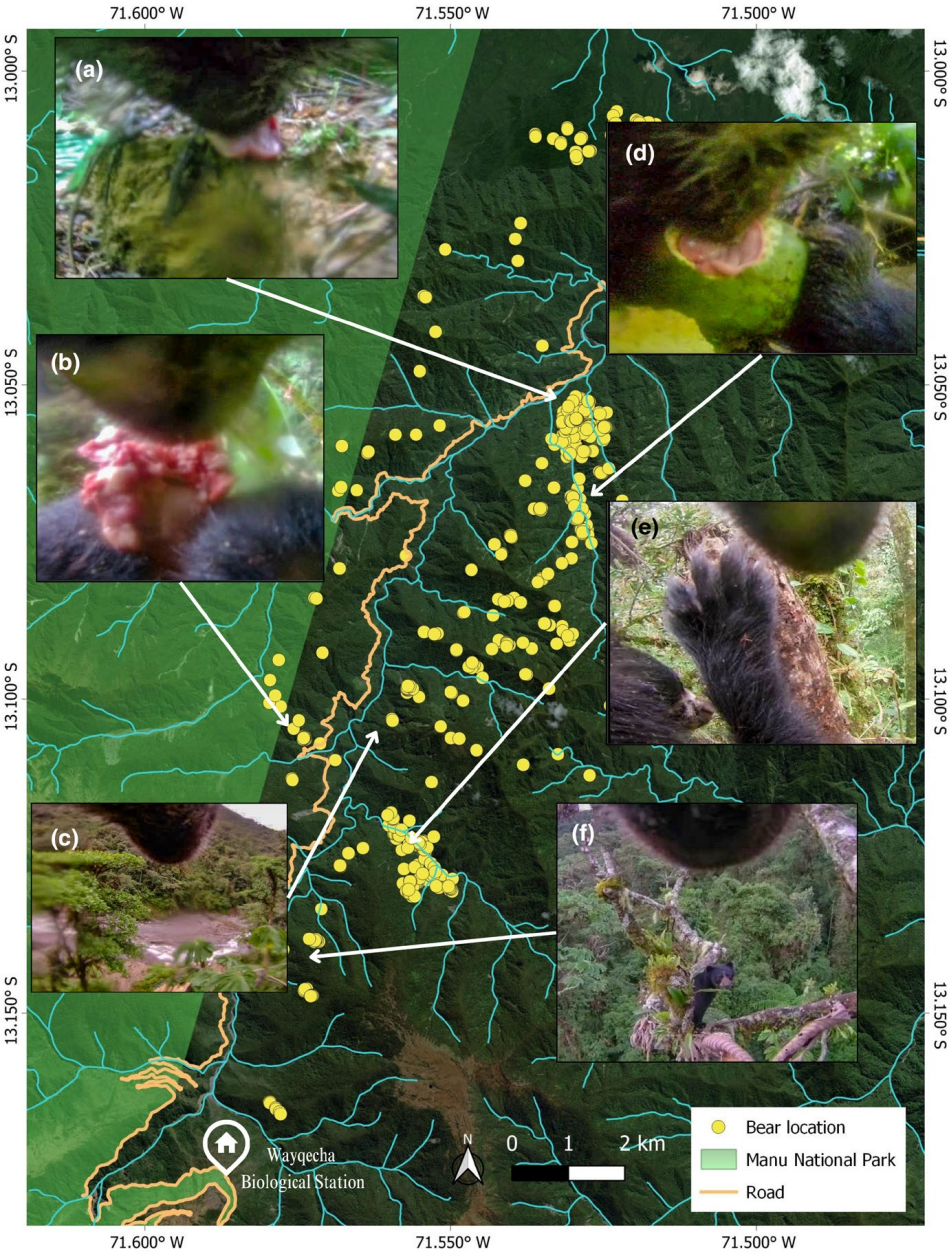
Map of the GPS locations recorded by the collared bear, accompanied by screen grabs documenting various events over the 4‐month period in the Kosñipata Valley. (a) Feeding on a clump of clay (geophagy) on January 10 at 4:00 pm (1360 m a.s.l.). (b) Feeding on the carcass of another Andean bear in the canopy on January 1 at 7:00 am (2198 m a.s.l.). (c) Observing the Kosñipata River from a canopy gap in the lower montane forest (1200 m a.s.l.) on December 31 at 8:00 am. (d) Feeding on *Porcella ponderosa* fruits on January 8 at 10:00 am (1731 m a.s.l.). (e) Mating with a female bear in the canopy on December 20 at 12:00 pm (2460 m a.s.l.). (f) Observing another bear in a fruit‐bearing *Cecropia* tree (2468 m a.s.l.). The arrows are indicating the approximate location where the footages were recorded.

One other video observation of note related to diet, is a single video clip of the collared bear eating soil/clay (see Figure 2). Geophagy is the consumption of soil and/or clay as some form of dietary supplement. Many species from the nearby Amazonian lowlands have been suggested to use “clay licks” for geophagy for a variety of possible uses (Griffiths et al., 2024): (1) As a potential mineral and nutrient supplement intake. (2) To detoxify secondary compounds from herbivory. And/or (3) As a sodium supplement. The western Amazon region in general is extremely sodium depauperate, as rains from the Atlantic have deposited any salt deposits way before reaching the Andean foothills. This means that herbivorous species in particular have to consume clay, usually from clay lick deposits exposed by eroding river banks or exposed pockets of sodium‐rich soil within the forest interior. However, there is a fourth potential explanatory factor. While geophagy has never been documented in Andean bears, brown bears in Yellowstone National Park (Mattson et al., 2016) and brown bears in the Russian Far East (Seryodkin et al., 2016) have both been found to display significant levels of geophagy, which has been attributed to helping prevent diarrhea. More observations and data will be required to understand if geophagy is generally used by Andean bears and if so, why.

### 3.2 Social interactions

The first instance of courtship and mating occurred over a period of 7 days, from December 18 to December 24, 2023. During this period, in a total of 21 clips, the collared bear was in the company of the same female bear (Female 1, previously identified in a camera trap through facial markings), engaging in activities such as socializing, sleeping, and mating. According to the collar GPS data, they remained in roughly the same location throughout this period. Mating behavior could be observed in no fewer than 10 video clips and occurred not only on the ground but up in the tree canopy on at least eight occasions. A second social event with a different female bear (Female 2) occurred on March 12, March 13, and March 17, 2024. The collared male interacted with a second female bear less frequently (just six clips). They interacted both in the trees and on the ground and remained roughly in the same location during this period, but no mating was recorded. This event took place 2 days before the collar was released, so no additional information was gathered. Knowledge about the mating behavior of the Andean bear in the wild is limited. Despite a low sampling size, our data support what has been observed in captivity where the female and male engage in courtship and copulation for approximately 7 days (García‐Rangel, 2012). Another elusive species, the Asiatic black bear, exhibits longer courtship periods, ranging from 9 to 22 days, also from similar collar technology (Naganuma et al., 2021). Most information on the Andean bear's courtship and mating season comes specifically from zoos, where mating occurs from February to September, depending on the photoperiod (García‐Rangel, 2012; Steyaert et al., 2020). In the wild, only sporadic records exist of direct observations and camera trap footage of bear pairs walking together in their natural habitat during the mating season. This occurs in December and January in the dry forest of Peru, following seasonal variation (Appleton et al., 2018). While in Chingaza and Huila, Colombia, records show mating activity in September and December (Infobae, 2021; Reyes et al., 2024). All of these records were made by terrestrially placed camera traps. As such, no records exist that involve mating in the canopy as we have documented here. Although we document mating and pairing behavior in both March and December, longer‐term data would be needed to confirm any specific seasonal breeding in southern Peru.

Although largely thought of as solitary animals (García‐Rangel, 2012), during the 4‐month period, in addition to the two female courtship interactions, the collared male also interacted with other bears on four occasions. The first instance occurred on January 4, as the collared bear was climbing down from a tree, a bear (unknown sex 1) could be seen among the lower branches, watching the collared bear before lowering its gaze. On a second occasion on January 14, while the collared bear is high up in a *Cecropia* tree, another bear (unknown sex 2) can be observed at a distance, sitting in a different *Cecropia* tree, observing one another (see Figure 2). On a third occasion on March 3, one bear (unknown sex 3) approaches the collared male in an aggressive manner and the collared bear is observed taking a defensive stance. Finally, in a fourth encounter on March 7, the collared bear appears a few meters above the ground in a tree, watching as another bear (unknown sex 4) passes through the bushes on the ground below. We could find no records in the literature on how Andean bears interact with individuals of their own species, except for females in estrus or mothers with their cubs (Appleton et al., 2018; Reyes et al., 2024). The only other published confirmation of sociality beyond breeding is a single record from Chingaza National Park in Colombia, where 28 scavenging events showed more than one bear feeding from the same carcass (Parra‐Romero et al., 2019).

## 4 CONCLUSIONS

In summary, the breadth of novel natural history information uncovered from just 4 months of camera collar data from a single individual of a poorly known species has elucidated many new insights and avenues of information that warrant further investigation. Andean bears are a challenging species to study, even when collared with traditional satellite GPS collars. This is because in many areas of their habitat, the terrain is so challenging that it is extremely difficult to locate individuals, even with the assistance of recent GPS data sent via satellite and VHF signals in the field. This means that getting eyes on the bears to directly observe what they are doing is immeasurably challenging. For other species that live in open conditions and can be reached by vehicle, aircraft flyovers, or tracked on foot, camera collars may prove less essential. However, animal‐borne camera collar devices, that have the potential to be released remotely via satellite when in a potentially retrievable location, could prove a game‐changing technology for hidden species of the tropics.

While many researchers might be deterred by the alarmingly high upfront costs of the collars (~$5000/unit vs. ~$2000 for a traditional GPS satellite non‐camera collar), when you consider the true capture costs of an animal (field staff and veterinarian salaries, field station costs, and other field logistics), which could easily amount to well over $30,000 with a team of four trained staff in a remote field station for 3 months, then the $3000 difference between collar types might not seem so off‐putting, especially given the added value from information that can be derived from the collars. Using such data to ground truth against GPS locations, and simultaneously gather signals in accelerometer data, provides the potential to build verified models in terms of activity or behaviors of the animals. This can then be applied to signals in accelerometer data gathered from traditional non‐camera collars. For example, recent research on polar bears has shown that combining a tri‐axial accelerometer with GPS cameras has allowed for the measurement of daily energy expenditure, as well as diet, behavior, and body composition in both land and water habitats (Pagano et al., 2017, 2024). These findings are essential for informing conservation efforts, particularly in the face of diminishing Arctic Sea ice and increasing polar bear land use.

Similarly, these technological advancements hold great potential for advancing our understanding of the Andean bear and its habitat. The integration of GPS and accelerometer data can provide invaluable insights into the behavior, natural history, and conservation needs of Andean bears, especially considering the threats posed by climate change and habitat loss. By leveraging these tools, researchers can develop more effective conservation strategies to mitigate human–bear conflicts and protect the Andean bear population and its habitat. To help with these challenges, it is critical that research funding resources are directed to those doing field surveys on poorly known wildlife species in the tropics. These are generally far less well studied than species residing in the global north, and tropical‐focused field teams need help to access the funds needed for the latest field technology, as funds within home countries are limited for such equipment. Another major assistance that could help empower science led by primary researchers based and working in low middle‐income countries could be for companies offering such technologies to offer discounted units under such circumstances.

Finally, we also highlight one major limitation of the collars at this current stage of their design—the lack of ability to gather data beyond daylight hours. Despite current battery consumption limitations, however, it is likely that infrared LED technology and battery advances will soon be able to tackle such barriers in years to come and allow researchers to gather a more complete diel insight into the lives of animals that are active during both day and night.

## AUTHOR CONTRIBUTIONS

**Ruthmery Pillco Huarcaya:** Conceptualization (lead); data curation (lead); funding acquisition (lead); investigation (lead); methodology (lead); project administration (lead); writing – original draft (lead); writing – review and editing (lead). **Andrew Whitworth:** Conceptualization (lead); data curation (lead); funding acquisition (lead); investigation (lead); methodology (lead); project administration (supporting); writing – original draft (lead); writing – review and editing (lead). **Norma Mamani:** Data curation (supporting); investigation (supporting); methodology (supporting). **Mark Thomas:** Investigation (supporting); methodology (supporting). **Elias Condori:** Investigation (supporting); methodology (supporting).

## CONFLICT OF INTEREST STATEMENT

The authors declare no conflicts of interest.

## Data Availability

Data from videos detailing the novel behaviors described in this paper are available on Figshare at https://figshare.com/s/44f8630669ce5933aa1b. A DOI will be provided upon manuscript acceptance. Similarly, Movebank data will be accessible and a DOI was obtained upon the manuscript's acceptance for publication.
